# Effect of weighted sled towing on sprinting effectiveness, power and force-velocity relationship

**DOI:** 10.1371/journal.pone.0204473

**Published:** 2018-10-05

**Authors:** Patrícia Dias Pantoja, Alberito Rodrigo Carvalho, Leonardo Rossato Ribas, Leonardo Alexandre Peyré-Tartaruga

**Affiliations:** 1 Exercise Research Laboratory, School of Physical Education, Physical Therapy and Dance, Federal University of Rio Grande do Sul, Porto Alegre, Brazil; 2 State University of Western Parana, Cascavel, Parana, Brazil; 3 Gymnastics Society of Porto Alegre (SOGIPA), Porto Alegre, Brazil; Nanyang Technological University, SINGAPORE

## Abstract

This study aimed to compare the components of force-velocity (F-V) and power-velocity (P-V) profiles and the mechanical effectiveness of force application (or force ratio–RF) among various sled-towing loads during the entire acceleration phase of a weighted sled sprint. Eighteen sprinters performed four 50-m sprints in various conditions: unloaded; with a load corresponding to 20% of the athlete’s body mass (BM); with a load of 30% BM; and with a load of 40% BM. Data were collected with five video cameras, and the images were digitised to obtain velocity from the derivation of the centre-of-mass position. F-V and P-V components and RF were estimated from sprinting velocity-time data for each load using a validated method that is based on an inverse dynamic approach applied to the sprinter’s centre-of-mass (it models the horizontal antero-posterior and vertical ground reaction force components) and requires only measurement of anthropometric and spatiotemporal variables (body mass, stature and instantaneous position or velocity during the acceleration phase). The theoretical maximal velocity decreased with load compared with the unloaded condition (for 20% BM: -6%, effect size (ES) = 0,38; for 30% BM: -15%, ES = 1.02; for 40% BM: -18%, ES = 1.10). The theoretical maximal horizontal force (F0) and maximal power were not different among conditions. However, power at the end of the acceleration phase increased with load (40% BM vs 0%: 72%; ES = 2.73) as well as the maximal mechanical effectiveness (12%; ES = 0.85). The linear decrease in RF was different between 30 or 40% BM and the unloaded condition (-23%; ES = 0.74 and 0.66). Better effectiveness may be developed with 40% BM load at the beginning of the acceleration and with the various load-induced changes in the components of the F-V and P-V relationships, allowing a more accurate determination of optimal loading conditions for maximizing power.

## Introduction

Acceleration is a determinant factor for success in sprinting events. Better sprint acceleration performance is associated with the athlete being able to exert a greater force in the horizontal direction, rather than applying a greater resultant force [1–3]. The orientation of the force applied by the athlete on the ground is evaluated with the effectiveness or ‘force ratio’, which is defined as the ratio of the horizontal force to the resultant force averaged over the stance phase [1]. A higher force ratio during acceleration is more desirable to achieve greater sprint performances. Studies have shown that an attenuated decrease in the force ratio during the sprint is highly correlated with a better performance [1, 2]. Therefore, a sprinter needs to direct the resultant force onto the ground horizontally and to limit the decrease in the force ratio along the acceleration phase of the sprint race.

Resistance training exercises, including sport-specific movements, are often used to improve sprint acceleration [4]. Training methods that add resistance to a sprint include wearing a weighted belt, towing a parachute, and towing a weighted sled [4–7]. Sled towing exerts a horizontal force on the athlete and is very effective in improving acceleration performance [4, 5, 8, 9]. In a sled-towing exercise, the athlete is attached to the sled through a chest or waist harness, and a series of sprints are performed with a specific weight added to the sled [4, 10]. The training stimulus depends mainly on the weight of the sled and the coefficient of sliding friction. The coefficient of friction must be considered because it influences the magnitude of horizontal frictional force between the sled and the running surface [11]. Studies of sled towing with different loads suggest that the athlete’s running speed should not be reduced by more than 10% because greater loads can induce detrimental changes in sprinting technique [5, 12]. However, heavy sled loads might be necessary to provide a sufficient training stimulus to increase force production and speed development during the acceleration phase of a sprint [10, 13–16].

Recent studies have examined the force-velocity relationship and power-velocity relationship during the acceleration phase of sprinting [17, 18]. The three main variables of these relationships are the theoretical maximal force (F_0_), the theoretical maximal velocity (V_0_) and maximal power (P_max_), which correspond to the maximal mechanical capabilities of lower limbs to produce external force, power and velocity. The athlete’s theoretical maximal velocity is obtained by extrapolation to zero force, and the theoretical maximal force is obtained by extrapolation to zero velocity. These parameters are influenced by the mechanical properties of muscles, neural activity, and joint configuration [2, 3, 19, 20]. Furthermore, the theoretical maximal force corresponds to the initial push of the athlete against the ground at the beginning of the acceleration phase. It is useful for sprinters and coaches to monitor this variable, since it reflects the athlete’s ability to transfer the overall strength at each lower-limb extension to the specific forward sprint motion at the first steps (if this transfer is good, then we would expect a good maximal force ratio) or at steps at high velocities (we would expect an increased ability to limit the decrease in force ratio) [18]. The theoretical maximal velocity reflects the sprint maximal velocity the athlete would be able to attain should mechanical resistances against movement be null [18]. It is associated with the capability to produce horizontal force at very high running velocities. The maximal mechanical power reflects the maximal capability of the athlete to produce the greatest F_0_ and V_0_ during sprint acceleration. Coaches should focus on increasing an athlete’s P_max_ by improving its components (F_0_ and V_0_), analyzing the horizontal profile of each athlete (horizontal force-velocity-power profile) which provide information about which underlying physical or technical feature is mainly limiting the performance. This allows for more individualized monitoring and training of physical and technical capabilities, respecting the characteristics of each athlete [18].

Therefore, these relationships are important because they inform about the athlete’s physical capabilities and if there is any imbalance in the capacity to produce force or velocity. For instance, two athletes with similar P_max_ could present different force-velocity profiles because of a different force/velocity combination. Maybe one athlete should focus more in training his or her force capability and another one should focus on training more his or her velocity capability. An optimal force-velocity profile will elicit a greater P_max_ and monitoring these relationships in sled towing training may be an effective method for coaches to stimulate an increase in the capacity for power production of an athlete, with an optimal load and an optimal force-velocity profile [2, 18]. A greater understanding of the effect of sled load on the force ratio during the acceleration phase of a sled-towing exercise might also be beneficial to athletes and coaches. For instance, if athletes are not directing their force in the optimum direction, or if the horizontal force decreases considerably during the acceleration phase, they might be able to use a sled-towing exercise to improve the orientation of their force application.

A study of ground reaction forces in the acceleration phase of a sled-towing exercise could use a 30–60 m long sequence of force plates [21]. However, such a system is very expensive. Recently, Samozino et al. [17] presented a simple method of determining the force-velocity relationship, power-velocity relationship, and force ratio in sprinting. The method showed excellent agreement with force plate measurements and is easy to use. It requires only measurement of anthropometric and spatiotemporal variables (i.e., body mass, stature, and instantaneous position or velocity during the acceleration phase of the sprint run). Furthermore, since the increasing load affects the entire trajectory of the sprint, it is necessary to understand these effects not just in the first seconds but also at the end of the acceleration phase. To the best of our knowledge, however, there are no studies analysing the mechanical power in this phase.

The aim of the present study was to examine the effect of sled load on the force ratio, the force-velocity relationship, and the power-velocity relationship during the acceleration phase of a sled-towing exercise. The primary hypothesis was that the force ratio would increase with increasing sled load due to the increasing horizontal force required to overcome the horizontal frictional force produced by the sled [22]. We also expected the power at the end of the acceleration phase to be greater in a sled-towing exercise than in unloaded sprinting.

## Materials and methods

### Participants and experimental protocol

Eighteen participants volunteered to be included in this study [12 men and 6 women; age: 18.4 ± 3.84 years (minimum: 13 years; maximum: 27 years); BM: 65.8 ± 11.3 kg (minimum: 47.3 kg; maximum: 83.1 kg); height: 1.70 ± 0.11 m (minimum: 1.51 m; maximum: 1.88 m); body fat: 12.2 ± 2.52% (minimum: 8.8%; maximum: 18.2%)]. The sample size was calculated in accordance with a previous study by Maulder et al. [14] investigating changes in sprint performance with resisted sled loading, with a statistical power of 80%. The participants were informed about the experimental procedures and gave their written informed consent. The study was in accordance with the ethical standards and with the Helsinki Declaration of 1975, as revised in 2008, and was approved by the local Ethics Committee of the Universidade Federal do Rio Grande do Sul. All participants were trained sprinters who were competing in 100 m or 400 m events (100 m: n = 13; 400 m: n = 5) and they were in the competition period of training. Their personal best times in the season was on average 11,8 ± 0,7 s on 100 m and 49,9 ± 6,5 s on 400 m (see reference [23] for IAAF points). Five athletes were of international level, five were of national level, and eight were of regional level. All participants were familiar with weighted sled towing. As inclusion criteria, athletes should be men or women training and competing in sprinting events for at least one year, they should be in their competition period (competing in regional, national or international events) and familiarized with sled towing. As exclusion criteria, athletes could not initiate during the study new physical activities that were not part of their normal routine; they should be free from any lower extremity injury that would prevent them from performing the tests; and they should not ingest drugs that could influence performance.

This study used a randomised crossover design and the experimental protocol comprised four 50-m sprints performed in four conditions: without a load, and towing a sled with 20, 30, and 40% body mass (BM). The mass of the sled was 5.2 kg and additional weights were placed on the sled to obtain the required load. The order of the trials was randomised and 15 minutes of passive rest were allowed between conditions. The participants were asked not to participate in physical exercise before the test. Before the test, the participants performed a 30-minute warm up consisting of dynamic stretching, jogging, technical drills, and submaximal sprints. All participants performed the test at the same time of day on an outdoor synthetic track consisting of polyurethane binders and rubber (Retorkan, Tartan™). Air temperature and wind speed did not differ substantially among the trials (<5%). Air temperature was measured with a thermometer positioned near the track, and wind speed was measured with a digital anemometer (ProSport, Santo Tirso, Portugal). The sled was attached to a waist harness by a 2.7 m cord (angle of tow cord = 19 ± 2°). A reliability test prior to the study showed that the sled towing protocol was reliable (ICC = 0.87–0.94). Pilot testing showed that a 50-m sprint distance was sufficient for all participants to reach their maximal velocity.

**Fig 1:**
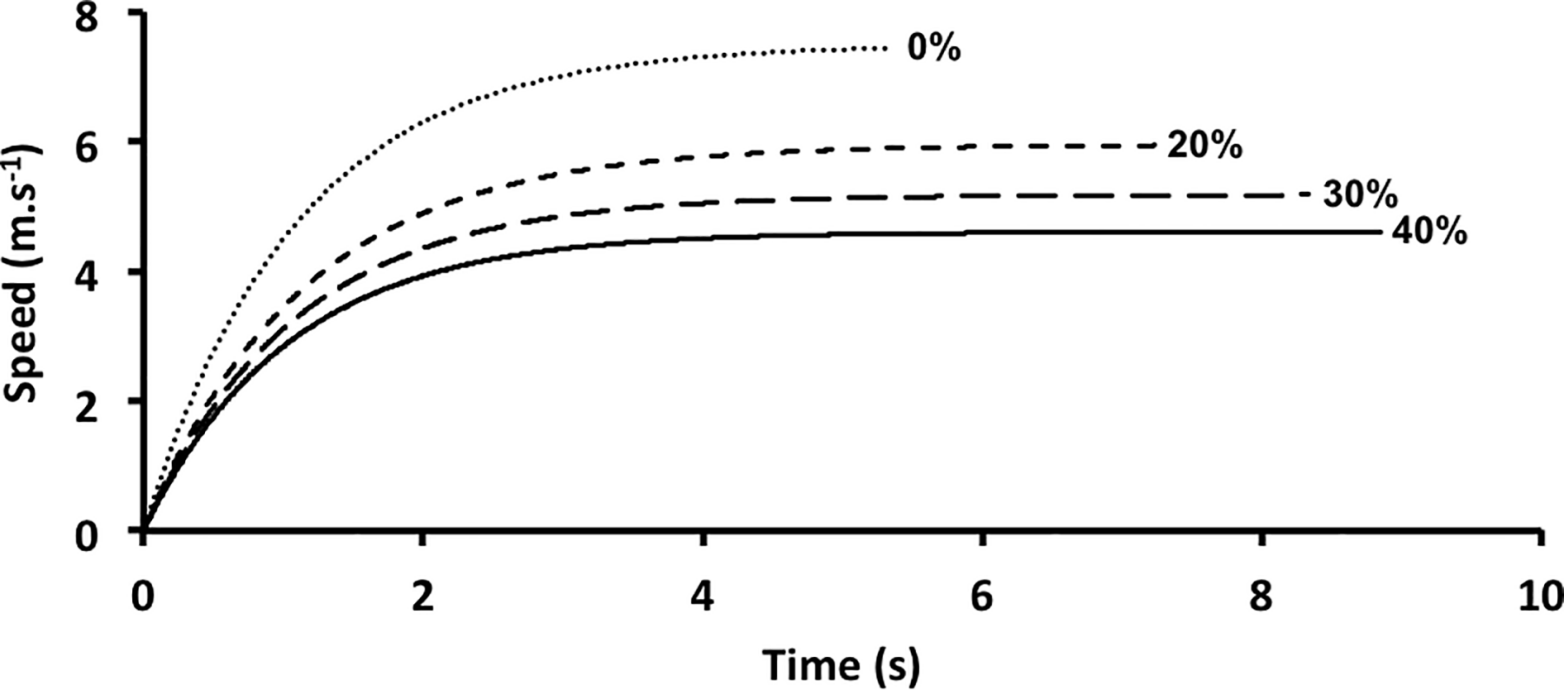
Horizontal velocity and acceleration as a function of time during a sprint acceleration phase, for each condition (percent of body mass). These data are from one representative individual.

The sprint trials were recorded using five high-speed (120 Hz) video cameras (CASIO EXILIM FH25, Tokyo, Japan) that were placed perpendicular to the sagittal plane of motion. The cameras had overlapping fields of view and each camera recorded about 10 m of the sprint. The distance between the cameras and the midline of the running lane was 10.54 m, and the average distance between cameras was 11 m. The five cameras were synchronised with LEDs that were positioned over the top of each camera lens and triggered at the same time. A device that produced sound and light when triggered was used to mark the start of the sprint trial. This study used automatic tracking of reflective markers. Nine reflective markers were placed on the participant over the following anatomical references: fifth metatarsal of the foot; heel; lateral malleolus; lateral condyle of the femur; greater trochanter; styloid process of the ulna; lateral epicondyle of the humerus; acromion of the scapula; and temporal bone. Two 2-m calibration rods with two reflective markers were recorded for each camera. The rods were placed so that one could be viewed in the next-nearest camera. Image reconstruction of the sprint trials was performed using the 2-D Direct Linear Transformation (DLT) method in SkillSpector software (1.3.2, Odense, Denmark). Position data were filtered using a fourth order Butterworth filter with a cut-off frequency of 3–8 Hz. The limbs on the side of the body opposite to camera did not have reflective markers. The locations of these limbs were estimated by applying a delay of 180 degrees to the camera-side limbs [24]. The location of the participant’s centre of mass was calculated from the anthropometric data for the following six segments: head-neck-trunk; forearm-hand; upper arm; thigh; shank; and foot [25]. The velocity of the participant’s centre of mass was obtained from the derivative of the centre of mass position. For each sprint trial, the velocity-time data were fitted with an exponential function where the fitted parameters were the maximal horizontal velocity and the time constant [17, 26, 27]. This function was used to decrease the noise and produce a smooth velocity-time curve (Fig 1). The acceleration-time curve of the participant’s centre of mass was obtained by taking the derivative of the equation for the velocity-time curve (Fig 1). The horizontal force on the participant was obtained by multiplying the acceleration-time curve by body mass plus the mass of the sled.

The force produced by the athlete was calculated as the sum of the resultant horizontal force on the participant, the aerodynamic drag force acting on the participant, and the horizontal force due to the sled [17]. The instantaneous aerodynamic force and friction force both depend on velocity. The aerodynamic force was estimated using the equation reported by Arsac and Locatelli [28], and the friction force was estimated using the equation reported by Cross et al. [11]. In the calculation of the friction force, the coefficient of sliding friction at slow speed was measured to be about 0.56, and the angle of the tow cord (θ) was obtained from video analysis using Kinovea software (v.0.8.15, Montceau-les-Mines, France).

The instantaneous power in the horizontal direction was calculated as the product of the participant’s force and velocity. Force-velocity and power-velocity plots for the participant were fitted with least-square linear and second-order polynomial regressions, respectively [29]. From the extrapolation of the force-velocity relationship to zero velocity and zero force, the values for F_0_ and V_0_ were obtained. The force ratio (RF) was computed as the ratio of horizontal ground reaction force to the corresponding resultant ground reaction force, i.e., technical ability of the athlete to orient the total ground reaction force vector forward [1]. The force ratio was plotted against time, and the slope of the linear decrease in force ratio (D_RF_) over the entire acceleration phase was calculated. The data before t = 0.3 s were not included in the calculations of RF and D_RF_ [1, 3, 17]. The maximal value of RF (RF_max_) was also obtained from t > 0.3 s.

Maximal power was calculated as P_max_ = (F_0_V_0_)/4 [19]. We also calculated power at the end of the acceleration phase, calculating the average value of the last second of the 50-m sprint. At that point, the participants were at their maximal velocity or near their maximal velocity (from 95% to 100%).

### Statistical analysis

Data are presented as mean ± standard deviation (SD) across all participants. Normality (Shapiro-Wilk test) was tested and an ANOVA for repeated measures was used to compare the four different loading conditions. If the distribution was not normal, a Friedman test was applied to compare the sprint conditions. A Bonferroni test was used to analyse the possible differences between loads. The 95% Confidence Interval was also obtained for each variable and the statistical procedures were performed using SPSS 24.0 (*p*<0.05). The effect size (ES) Cohen’s *d* coefficient was also calculated to assess the magnitude of differences. The criterion for interpreting ES was: trivial (< 0.2), small (0.2–0.6), moderate (0.6–1.2), or large (> 1.2) [14, 30, 31]. We also obtained the standardized effect sizes of the ANOVA tests.

## Results

**Fig 2:**
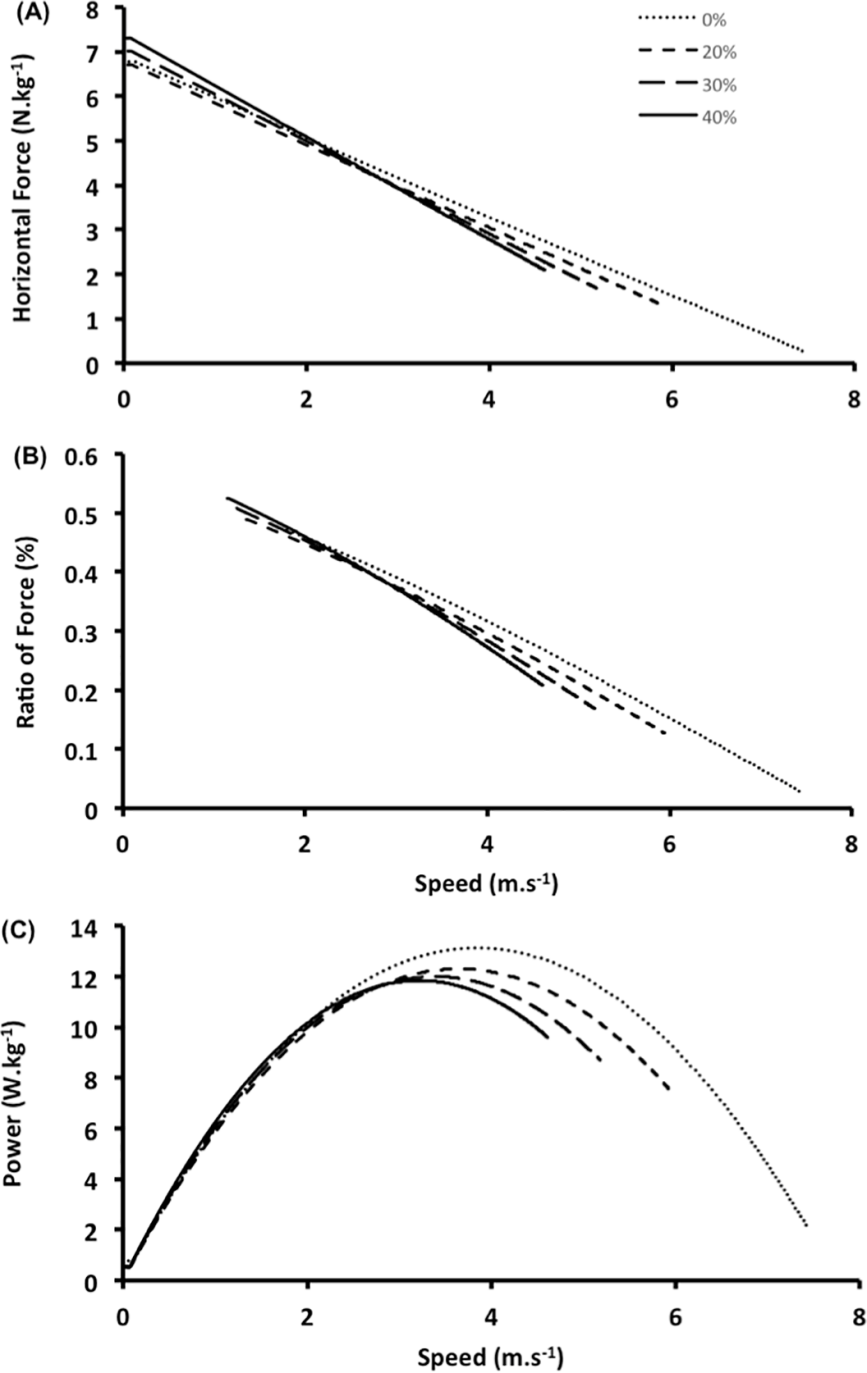
The key mechanical parameters of sprint as a function of speed. Conditions: 0%- unload, 20%, 30%, 40% of body mass. (A) Force-velocity relationship. (B) Ratio of force (*RF*) as a function of speed. The *D*_*RF*_ index is the slope of the decrease in *RF* with speed (in this figure: 0% = -0.085; 20% = -0.085; 30% = -0.091; 40% = -0.084). (C) Power-velocity relationship. These data are from one representative individual.

Mechanical variables for each load are shown in Table 1 (figures corresponding to these data are added as supplementary material–S1 Fig). Repeated-measures ANOVA revealed a significant load effect for maximal velocity (V_0_; ES = 0.51; p < 0.001), maximal force ratio (RF_max_; ES = 0.37; p = 0.002), decrease in force ratio (D_RF_; ES = 0.25; p = 0.013) and sprint time (ES = 0.93; p < 0.001). However, there was no clear effect of sled load on the maximal force (F_0_; ES = 0.15; p = 0.059; Fig 2A) or maximal power (P_max_; ES = 0.11; p = 0.164; Fig 2C). The maximal velocity decreased with increasing sled load (ES = 1.02 and 1.10 for 30% and 40% BM, moderate effect). The maximal force ratio increased with increasing sled load (ES = 0.57–0.87, small to moderate effect), and the decrease in the force ratio during the acceleration phase was greater with increasing sled load (ES = 0.74 and 0.66 for 30% and 40% load, moderate effect). The 50-m sprint time showed a substantial increase with the increasing sled load (ES = 1.64–2.99, large effect).

**Table 1:** Sprint running mechanics during the entire sprint acceleration. Data are presented as mean *(SD)* and 95% confidence intervals are reported for each load (0%; 20%; 30%; 40%).

	Mean *(SD)*		95% CI
**Theoretical maximal velocity *V***_***0***_ **(m.s**^**-1**^**)**			
**0%**	7.35	*(1*.*08)*	6.74–7.94
**20%**	6.93	*(1*.*10)*	6.28–7.32
**30%**	6.26	*(1*.06)* †	5.70–6.86
**40%**	6.04	*(1*.28)* †	5.37–6.76
**Theoretical maximal horizontal force *F***_***0***_ **(N.kg**^**-1**^**)**			
**0%**	8.29	*(2*.*29)*	6.94–9.41
**20%**	8.62	*(1*.*87)*	7.67–9.71
**30%**	9.49	*(2*.*43)*	8.05–10.64
**40%**	9.52	*(2*.*82)*	7.67–10.53
**Computed maximal power output *P***_***max***_ **(W.kg**^**-1**^**)**			
**0%**	15.0	*(3*.*93)*	12.5–16.9
**20%**	14.7	*(2*.*85)*	13.0–16.0
**30%**	14.5	*(3*.*22)*	12.6–16.1
**40%**	13.9	*(3*.*70)*	11.5–15.2
**Computed power output at the end of the acceleration phase P**_**ea**_ **(W.kg**^**-1**^**)**			
**0%**	2.68	*(1*.*72)*	1.83–3.54
**20%**	7.43	*(2*.49)*	5.94–8.27
**30%**	8.69	*(1*.97)*	7.62–9.79
**40%**	9.46	*(3*.06)* † ◊	7.94–10.98
**Computed percentages of RF**_**max**_ **(%)**			
**0%**	49.8	*(8*.*3)*	44.7–53.9
**20%**	53.9	*(6*.04)*	50.5–57.2
**30%**	56.3	*(6*.48)* †	52.5–59.4
**40%**	56.7	*(8*.02)* †	51.4–59.7
**Computed decrease in the ratio of force D**_**RF**_			
**0%**	- 0.106	*(0*.*034)*	-0.123 –-0.086
**20%**	- 0.112	*(0*.*036)*	-0.133 –-0.096
**30%**	- 0.137	*(0*.049)*†	-0.162 –-0.108
**40%**	- 0.137	*(0*.057)*	-0.161 –-0.102
**Time 50 m (s)**			
**0%**	6.78	*(0*.*65)*	6.43–7.11
**20%**	8.06	*(0*.89)*	7.61–8.52
**30%**	8.94	*(1*.13)* †	8.32–9.52
**40%**	9.91	*(1*.33)* † ◊	9.17–10.57

* significantly different from 0% load.

† significantly different from 20% load.

◊ significant difference between 30% and 40%.

Repeated-measures ANOVA also revealed a significant load effect for power at the end of the acceleration phase (P_ea_; ES = 0.89; p < 0.001). It increased substantially with increasing load (ES = 2.22–3.25, large effect, compared to unloaded sprint). P-V, F-V and RF-V relationships are shown in Fig 2.

## Discussion

The main findings of this study are as follows: (1) maximal mechanical effectiveness of force application increased with load, and the ability to limit the drop in effectiveness during the sprinting acceleration phase (i.e., to maintain RF as much as possible) decreased when towing a sled with 30% or 40% BM compared with the unloaded condition; and (2) power computed at the end of the acceleration phase was greater with increasing load. It is worth noting that this study included sled-towing loads (30 and 40% BM) that were heavier than the recommended load of less than 20% BM [5].

We accept our first hypothesis, as expected, that the effectiveness or technical ability of force application is enhanced with the increasing load. This raises the question of whether a training protocol using sled towing may change the force direction positively in the sprint. The ES between the control condition and 40% BM (0.85) for RF_max_ was higher than between the unloaded condition and 20% BM (0.57). This is in accordance with studies addressing the use of heavier loads because lighter loads would not provide sufficient stimulus to develop sprint performance [10, 15, 32]. Furthermore, the RF-V relationship for the entire acceleration phase presented in Fig 2B shows that the effectiveness is greater for heavier loads at the beginning of the acceleration phase until a point where the difference between the conditions appears to be reduced. Therefore, using weighted sled towing (up to 40% BM) may be a useful and practical method for sprinters to develop their speed by improving their technique of orienting the force application in a more horizontal direction, especially at the beginning of the acceleration phase. Indeed, the F-V relationship presented in this study (Fig 2A) shows that the horizontal force was higher at the beginning of the acceleration phase, with less difference between loading conditions at the beginning than at the end of the acceleration phase where the horizontal force was lower for the loaded conditions. Therefore, the horizontal force may contribute more to the greater effectiveness (force ratio) of heavier loads at the beginning of the acceleration phase. However, if the athlete’s goal is to develop power resistance, then is probably interesting to use the sled loads during the entire acceleration phase.

The sprinters reduced the effectiveness of applying force to the ground in a horizontal direction (D_RF_) during sprinting while towing a sled with heavier loads. To our knowledge, this computation has not been performed previously for various sled-towing conditions in sprint running. Morin et al. [2] investigated the mechanical determinants of 100-m sprinting and found a significant correlation between the D_RF_ index and sprint performance. Possibly, with heavier loads than the ones used in this study, the D_RF_ index may be even steeper. This index may be a good representative of the technical ability of a sprinter during the entire acceleration phase, and therefore, it may be an interesting parameter for the evaluation of the effect of training with resisted sled sprinting. We suggested that after training with a determined sled load, the sprinter will be better able to limit the decrease in RF and improve the performance by orienting better the application of force in a forward direction during a greater proportion of the acceleration phase. Additional studies are needed to validate these observational findings and to further define optimal loads for sled training for sprinters.

Our results also showed that towing a weighted sled with various loads had no significant effect on horizontal F_0_. Kawamori et al. [16] investigated the GRF of the second ground contact after the start of a 5-m sprint towing a weighted sled with loads from 10 to 30% BM. The authors found a higher force ratio and net horizontal impulse for the load corresponding to 30% BM compared with the unloaded condition. It was suggested that the greater net horizontal impulse was probably due to a longer contact time rather than greater force production (similar horizontal GRF between the 30% condition and the unloaded condition). These findings agree with those of the present study, since F_0_ was not different among sled-towing conditions, suggesting that a greater magnitude of horizontal force application may not be the main determinant for the greater force ratio observed with higher loads. At the start of sprinting, there is a great need to increase the kinetic energy from zero to maximal speed. Therefore, a high F_0_ is necessary at the beginning of the sprint for the unloaded and loaded conditions, to accelerate the body forward, with little difference between them. In addition, it is possible that a longer contact time with higher loads plays a more important role, as claimed by Kawamori et al. [16]. The optimal combination of contact time and horizontal force production may be important for improvements in the force ratio and sprinting performance.

Our second hypothesis was also confirmed: mechanical power increases more critically at the end of the acceleration phase (the last second of the 50-m sprint) than at the initial phase, when towing a weighted sled. Some important details deserve comment. First, the greater mechanical power at the end of acceleration with the sled, (as opposed to the unloaded condition) may be due to increased resistance of the device to propel the body forward in this phase. This finding is presumably due to basic differences in the mechanical work produced during the entire acceleration. At the start, there was a great need to increase the kinetic energy from zero to maximal speed, whereas the work done to reaccelerate the body forward was extremely reduced at the final acceleration [26, 33]. Therefore, the constant level of horizontal external force transmitted to the sprinter’s waist played a more important role in mechanical power generation during the second half of the acceleration. Also, force friction was increased with heavier loads, and the coefficient of friction was negatively related to sprinting velocity, reaching a peak until around 5 m.s^-1^ [11]. Hence, it is possible that our athletes experienced greater resistance as they approached their maximal velocity sprinting with heavier sled-towing loads and were thus performing more work, which resulted in the observed higher power output at the end of the acceleration phase. This parameter may be useful in determining an optimal load for resisted sled sprinting, i.e., the load that will elicit the highest power [34]. Interestingly, the average theoretical and experimental maximal velocity found out in our study (7.35 ± 1.08 m.s^-1^) was lower than the results from another study (9.55 m.s^-1^) [34]. It is important to point out that factors such as competition period of training and fear of injury may have influenced these findings.

The study has some limitations that must be addressed. The athlete group used here was heterogeneous since men and women were recruited and participants had different sprint specialties (five were competing in 400-m events). However, all had similar training and were used to training at all sprinting distances. We included all individual responses in the supplementary material (S1 Table). Moreover, we acknowledge the fact that prescribing a sled load based on % BM does not consider any possible variation in individual strength or power among the participants. However, the main mechanical responses from different loads could be observed. During the period in which this study was conducted, there were no more appropriate validated methods for prescribing individual sled loads. It should also be noted that the model used in our study [17] has limitations such as estimating the horizontal aerodynamic drag force from only stature, body mass and a fixed drag coefficient [28], as well as having the assumption of a quasi-null centre-of-mass vertical acceleration during the sprint (variables are step-averaged, i.e., it includes contact plus aerial times). Nevertheless, Samozino et al. [17] found in their study force-modeled values that were very close to values measured by force plates. For instance, vertical force values were very close to body weight when averaged over 40 m of sprinting acceleration. Furthermore, we believe that the behaviour of the *F-V* and *P-V* relationships when comparing various sled loads in this study was not affected by the methods used, since it was in agreement with other studies evaluating these parameters.

## Conclusions

The study demonstrated that the orientation of force application in the horizontal direction (force ratio) increases with load, indicating that heavier loads permit the sprinter to apply the resultant force more horizontally. Therefore, training with sled loads may improve the ability of the sprinter to apply the resultant force in a horizontal direction, during the acceleration phase. This result is crucial for coaches planning to use the sled-towing method to train their athletes. Force ratio should be a useful marker to analyse the effect of training (to detect if the sprinter is increasing the ability to orient his or her force application more horizontally) and to determine when a new load should be applied. Another finding in this study was that *P*_*ea*_ increased with load. Heavier loads may be also used to enhance the generation of power at the end of the acceleration phase for sprinters.

## Supporting information

S1 Fig Mean and standard deviation of mechanical parameters at different sled-towing loads (0%; 20%; 30%; 40% of body mass). Theoretical maximal velocity (*V*_*0*_, A), theoretical maximal force (*F*_*0*_, B), computed maximal power (*P*_*max*_, C), computed power output at the end of the acceleration phase (*P*_*ea*_, D), computed ratio of force (*RF*_*peak*_, E), computed decrease in the ratio of force (*D*_*RF*_, F), and time of 50-m sprint (time 50-m, G) * significantly different from 0% load. *† significantly different from 20% load*. ◊ *significant difference between 30% and 40%*.
(TIFF)

S1 Table General dataset. (XLSX)
