# Supplementary figures and images for: Effect of weighted sled towing on sprinting effectiveness, power and force-velocity relationship
Source: PLoS One. 2018 Oct 5;13(10):e0204473. doi: 10.1371/journal.pone.0204473 (PMC6173386; doi:10.1371/journal.pone.0204473)

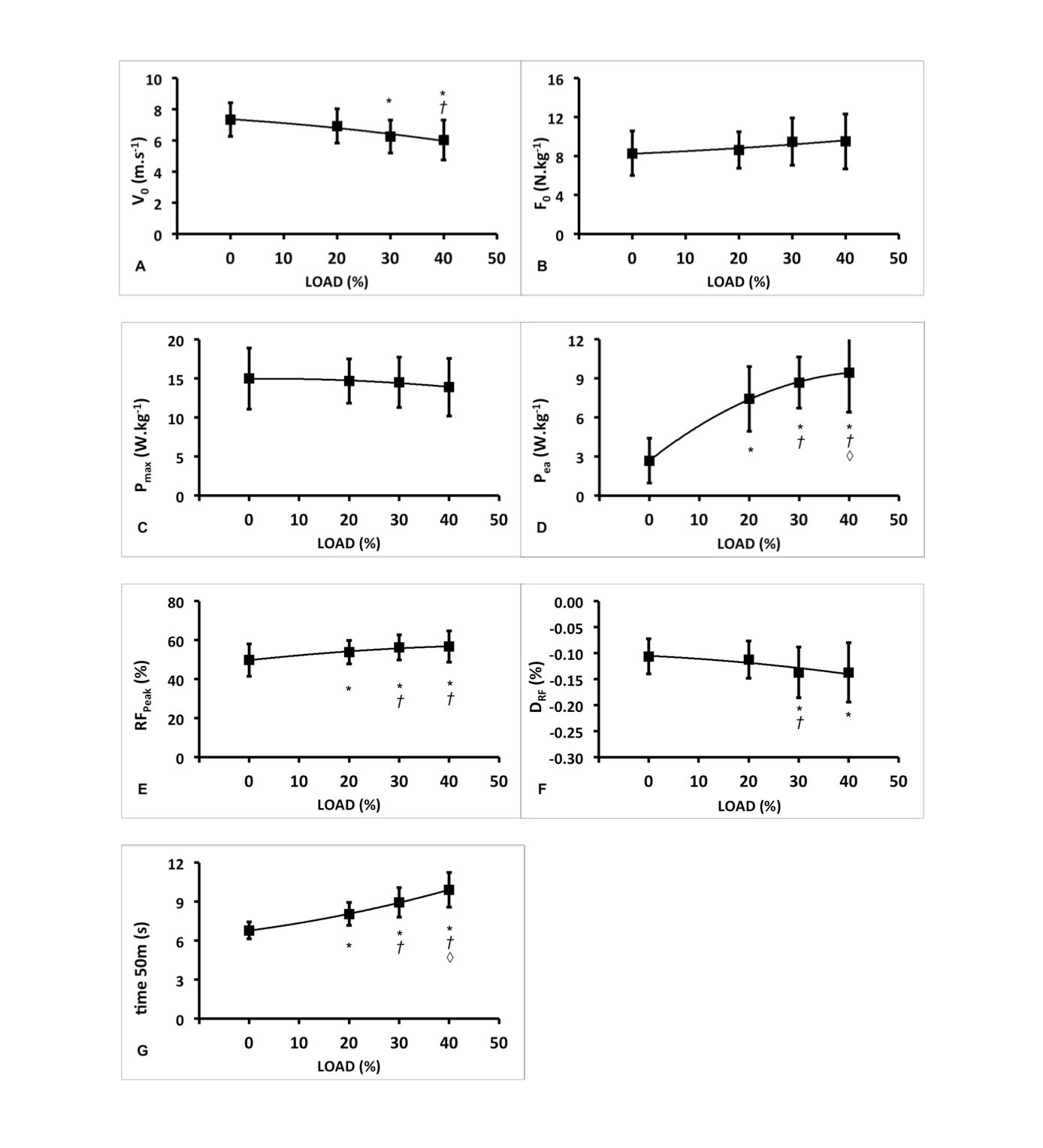

Supplement: S1 Fig — Theoretical maximal velocity (V0, A), theoretical maximal force (F0, B), computed maximal power (Pmax, C), computed power output at the end of the acceleration phase (Pea, D), computed ratio of force (RFpeak, E), computed decrease in the ratio of force (DRF, F), and time of 50-m sprint (time 50-m, G) * significantly different from 0% load. † significantly different from 20% load. ◊ significant difference between 30% and 40%. (TIFF) [file pone.0204473.s001.tiff]
